# Root inoculation with soil‐borne microorganisms alters gut bacterial communities and performance of the leaf‐chewer *Spodoptera exigua*

**DOI:** 10.1111/1758-2229.70049

**Published:** 2024-11-26

**Authors:** Beatriz Ramírez‐Serrano, Marina Querejeta, Zhivko Minchev, María J. Pozo, Géraldine Dubreuil, David Giron

**Affiliations:** ^1^ Biodiversity and Interactions Between Micro‐organisms/Insects/Plants (IMIP) Institut de Recherche sur la Biologie de l'Insecte (IRBI)—UMR 7261 CNRS/Université de Tours Tours France; ^2^ Department of Soil and Plant Microbiology Estación Experimental del Zaidín (EEZ‐CSIC) Granada Spain; ^3^ UMR CNRS 7267, Ecologie et Biologie des Interactions Université de Poitiers Poitiers France; ^4^ Agronomical Development Department, Business Unit Microbiology Koppert Biological Systems Berkel en Rodenrijs The Netherlands

## Abstract

Soil‐borne microorganisms can impact leaf‐chewing insect fitness by modifying plant nutrition and defence. Whether the altered insect performance is linked to changes in microbial partners of caterpillars remains unclear. We investigated the effects of root inoculation with soil bacteria or fungi on the gut bacterial community and biomass of the folivore *Spodoptera exigua*. We also explored the potential correlation between both parameters. We performed herbivory bioassay using leaves of tomato plants (*Solanum lycopersicum*), measured caterpillar weight gain and characterized the gut bacterial communities via 16S rRNA gene metabarcoding. All soil microbes modified the gut bacterial communities, but the extent of these changes depended on the inoculated species. *Rhizophagus irregularis* and *Bacillus amyloliquefaciens* had opposite effects on *S. exigua* weight. While plant inoculation with the fungus influenced gut bacterial diversity, *B. amyloliquefaciens* also affected the community composition. A reduced abundance of two *S. exigua* enterococcal symbionts correlated with decreased insect biomass. Our results show that soil microorganisms can induce plant‐mediated changes in the gut bacterial community of foliar‐feeding caterpillars. We propose that the impact of these alterations on insect performance might rely on specific adaptations within the gut bacteria, rather than solely on the occurrence of changes.

## Graphical abstract

Root inoculation with soil bacteria or fungi alters the gut bacteria of a generalist folivore caterpillar, with different inoculated species producing varying effects on insect biomass. These changes occur despite the absence of direct contact between the microbially treated plant compartment and the plant shoot used to feed the herbivore. Correlations suggest that specific microbial adaptations within the gut can influence larval performance.

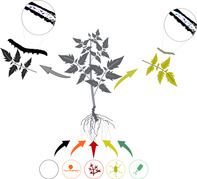

## INTRODUCTION

Plants are primary providers of resources for numerous aboveground and belowground organisms and play a central role in their interactions (Koricheva et al., 2009; Pineda et al., 2015; Rasmann et al., 2017). Association with rhizospheric microorganisms can modify nutritional and defensive plant traits, affecting the performance of insect herbivores that feed on them (Gruden et al., 2020; Harun‐Or‐Rashid & Chung, 2017; Song et al., 2013). They can protect plants against herbivores when used as bioinoculants, representing a promising alternative to the intensive inputs of agrochemicals (Kergunteuil et al., 2016; Pineda et al., 2010; Poveda, 2021). However, plant–microbe–insect interactions are influenced by several abiotic and biotic factors and regulatory mechanisms, driving to different outcomes (Pozo et al., 2020). Our limited understanding on these mechanisms is a major obstacle for the optimization and wider adoption of microbial‐based products for crop protection (Gruden et al., 2020; Lee Díaz et al., 2021; Trivedi et al., 2017).

Arbuscular mycorrhizal fungi (AMF), *Trichoderma* spp. and plant growth‐promoting rhizobacteria (PGPR) from the genera *Bacillus* and *Pseudomonas* have been well‐studied providers of benefits for plant health (Berg, 2009; Borriss, 2011; Guzmán‐Guzmán et al., 2019; Jeffries et al., 2003; Lugtenberg & Kamilova, 2009; Sood et al., 2020). They can promote plant growth through facilitation of nutrient acquisition (i.e., phosphate solubilization or iron sequestration) or by modulation of phytohormone levels (Begum et al., 2019; Berg, 2009; Gruden et al., 2020; Hayat et al., 2010; Nadeem et al., 2014; Richardson et al., 2009). Plant beneficial microbes can also enhance the plant defensive capacity, inducing resistance against a wide range of attackers (Palermo et al., 2022; Pieterse et al., 2014; Pineda et al., 2010; Poveda, 2021; Pozo & Azcón‐Aguilar, 2007; Ramamoorthy et al., 2001). Association with beneficial microbes can boost both direct and indirect mechanisms of plant defence to counteract herbivory (Pineda et al., 2010; Poveda, 2021; Rasmann et al., 2017). Direct mechanisms often target insect's gut through anti‐nutritional or toxic effects (Chen, 2008; Felton, 2005; Howe & Jander, 2008; Konno & Mitsuhashi, 2019; War et al., 2012). Plants can also limit the food supply (i.e., reinforcing cell walls or synthetizing repellent compounds) or reduce plant nutritional value to insects, causing pre‐ or post‐ingestion anti‐nutrition, respectively (Chen, 2008). Plant beneficial microbes can also enhance nutrient acquisition, increasing plant nutritional value, and they can help plants to overcome biomass losses caused by herbivores via compensatory mechanisms (Bennett & Bever, 2007; Zeng et al., 2022). Thus, the impact of plant‐associated microorganisms can range from negative, no effect or even positive effects on phytophagous performance (Shikano et al., 2017).

Among the order Lepidoptera, there are multiple examples of polyphagous and widespread pests that feed on leaves, roots or fruits of relevant crops causing important yield losses (Southon et al., 2019; Zheng et al., 2011). Chemical pesticides have been traditionally used for caterpillar management in agricultural systems, but continuous and prolonged applications have triggered the emergence of resistant populations (Aldosari et al., 1996; Che et al., 2013; Wang et al., 2021) and have undesirable effects on their natural enemies, other animals, and even on human health (Liu et al., 2016; Ruberson et al., 1994; Zheng et al., 2011). Therefore, there is an increasing social demand for effective sustainable alternatives to control insect pest populations while reducing the risks associated to chemical insecticides usage.

In general, gut bacterial communities of lepidopterans are variable but simple (Douglas, 2015; Hammer et al., 2017; Voirol et al., 2018). They are proposed to aid to digest the plant tissues and facilitate nutrient acquisition (Pinto‐Tomás et al., 2011; Visôtto, Oliveira, Guedes, et al., 2009; Xia et al., 2017), to suppress plant anti‐herbivore responses (Acevedo et al., 2017) or minimize harmful effects of plant allelochemicals (Hammer & Bowers, 2015; Visôtto, Oliveira, Ribon, et al., 2009; Xia et al., 2017). For instance, plant defences can target the peritrophic membrane that protects caterpillar midgut from pathogen colonization and compartmentalize the digestive processes (Konno & Mitsuhashi, 2019; Pechan et al., 2002). Gut bacterial communities can contribute to the maintenance of integrity and adequate permeability of this structure (Hegedus et al., 2008; Rodgers et al., 2017; Song et al., 2018; Zha et al., 2021). Several studies have demonstrated that gut microbiota manipulation adversely affects caterpillar fitness (Broderick et al., 2006; Chen et al., 2020, 2022; Wang et al., 2020). This topic remains contentious, as other studies report no discernible effect on germ‐free or antibiotic‐treated caterpillars (Devi et al., 2022; Raymond et al., 2009). Diet is suggested as one of the most important factors influencing the gut bacterial assemblages in lepidopterans (Jones et al., 2019; Martínez‐Solís et al., 2020; Mason et al., 2020; Mason & Raffa, 2014; Tang et al., 2012), but how specific nutritional and defensive plant traits contribute to the differences observed remains poorly understood. Within the same host plant, variations in certain plant nutritional parameters affect (Hu et al., 2022) or not (Ramírez‐Serrano et al., 2022) the gut bacterial communities of caterpillars. Contradictory observations are also reported when evaluating the effects of specific plant defensive compounds on caterpillar microbiomes (Gasmi et al., 2019; Ramírez‐Serrano et al., 2022).

To date, only few studies have investigated the impact of soil microorganisms—as assemblages of several species (Hannula et al., 2019) or as a single microbial species (Di Lelio et al., 2023)—on the microbiota of leaf‐chewing caterpillars. Thus, despite increasing research efforts, the individual contribution of soil microorganisms to shaping the bacterial community of caterpillars and its possible connection with larval performance remains unclear. In this study, we aimed at shedding light onto the effect of root inoculation with different single microbial species on larval gut bacterial communities and its possible relationship with the growth of insects fed on these plants. We hypothesized that soil beneficial microbes can modify the gut bacterial community of foliar‐feeding caterpillars via plant‐mediated effects. If so, these changes can impact caterpillar performance. To test our hypothesis, we conducted an herbivory bioassay using tomato (*Solanum lycopersicum*) root‐inoculated with different single plant beneficial bacterial or fungal species and *S. exigua* caterpillars. Larvae were fed on detached leaves for several days and their weight was recorded. Then, 16S rRNA gene metabarcoding was used to reveal potential differences in the gut bacterial communities of the larvae. This study has the final aim of enhancing our understanding on how microorganisms of different trophic levels can contribute to the final outcome of plant microbial symbiont–plant–insect interactions.

## EXPERIMENTAL PROCEDURES

### Microbe growing conditions and inoculum preparation

One species of AMF, *Rhizophagus irregularis* (Ri), the biocontrol fungus *Trichoderma afroharzianum* T22 (T22) and two PGPR, *Bacillus amyloliquefaciens* (Ba) and *Pseudomonas azotoformans* (Pa), were used to investigate the effects of soil microbes on the bacterial community and performance of *Spodoptera exigua*. A non‐inoculated (Ni) treatment was also included as control. Each species was used in monoassociation with tomato (*Solanum lycopersicum*) plants.

*Rhizophagus irregularis* MUCL 57021 was obtained from a monoxenic culture. *Agrobacterium rhizogenes*‐transformed roots of carrot (*Daucus carota*) on minimal (M) medium were used to host the AMF (St‐Arnaud et al., 1996). To dissolve the agar and extract the spores, 3:1 (v/v) of citrate buffer 0.01 M (pH = 6) was added to the sporulating culture and mixed in a rotary shaker for 1 h. The spores were then recovered using sieves (250 and 53 μm), re‐suspended in sterile water at final concentration of 1000 spores/mL (Minchev et al., 2021).

*Trichoderma afroharzianum* T22 was grown on potato dextrose agar (PDA, Difco) for 7 days at room temperature. Spores were collected in sterile tap water and the concentration of the spore suspension was quantified using a Bürker‐Türk counting chamber. Concentration was then adjusted to 1 × 10^7^ spores/mL (Minchev et al., 2021).

*Bacillus amyloliquefaciens* CECT8238 were cultured in tryptone soya agar (TSA, Oxoid) for 24 h at 28°C. A single colony was isolated from the TSA culture, inoculated in 25 mL of Difco sporulation medium (DSM; Nicholson & Setlow, 1990) and incubated for 48 h at 28°C in a rotatory shaker (200 rpm). The spore concentration was quantified with a Bürker‐Türk counting chamber and centrifuged at 5000 rpm for 15 min. Then, they were re‐suspended in sterile water to a final concentration of 1 × 10^7^ spores/mL (Minchev et al., 2021).

*Pseudomonas azotoformans* F30A was cultured on TSA for 24 h at 28°C. Liquid pre‐culture was prepared using tryptone soya broth (TSB, Oxoid) inoculated with a single bacterial colony and incubated overnight at 28°C with rotary shaking at 200 rpm. 25 mL of TSB media was inoculated with 1 mL of pre‐culture and placed in a rotatory shaker (200 rpm) at 28°C. After 150 min of incubation, with bacterial growth in exponential phase, the cell concentration was calculated measuring the O.D. (620 nm). The bacterial culture was centrifuged at 5000 rpm for 15 min, and the bacterial cells were re‐suspended in sterile tap water to a final concentration of 1 × 10^7^ cfu/mL (Minchev et al., 2021).

Plants treated with either Ri, T22, Ba or Pa were inoculated with 1 mL of the corresponding inoculant (Minchev et al., 2021), and 1 mL of water was added to the roots of Ni control plants.

### Plant material and growing conditions

Seeds of *S. lycopersicum* cv Money maker (Vreeken's Zaden, The Netherlands) were surface sterilized by immersion in 5% sodium hypochlorite for 10 min. Then, seeds were washed three times with sterile water for 10 min. Surface‐sterilized seeds were sown in sterile vermiculite and incubated for 7 days in a greenhouse at 24°C: 16°C day: night with 16 h: 8 h light: dark of photoperiod and 60% of relative humidity. Tomato seedlings were transferred to 300 mL pots that contained a mixture of gamma‐irradiated nutrient‐poor sandy soil (BVB, The Netherlands) and sterile vermiculite (1:1) and inoculated with the microbial treatments as previously described. Once inoculated, the plants were randomly distributed in a greenhouse and grown for 6 weeks at 24°C: 16°C day: night with a photoperiod 16 h: 8 h light: dark and 60% of relative humidity. Plants were watered twice per week with Hewitt nutrient solution (Hewitt, 1966) reduced in phosphorous (0.67 mM, 50% of the standard concentration) to ensure mycorrhizal establishment.

### Microbial detection in plant roots

At the end of the experiment, the detection of the inoculated microbes in root samples was assessed through qPCR. DNA from roots was extracted using a DNA extraction kit (Xtrem Biotech, Spain) following the manufacturer's instructions. DNA was quantified using NanoDrop (Thermo Fisher Scientific United States) and diluted to an equal concentration of 10 ng/μL for all samples. qPCR was performed using the commercial kit SYBR Premix Ex Taq Perfect Real Time (Takara, Japan) and the StepOnePlus™ Real‐Time PCR System (Applied Biosystems, United States). For the detection of DNA of the inoculated microbes, we used species‐specific primers (Table S1) for each of the microbes used in this study and the amount of microbial DNA was referred to the tomato plant DNA.

The presence of all inoculated microbes was confirmed in their corresponding treatments (Table S2) and their absence in the non‐inoculated control treatment.

Mycorrhizal colonization also was assessed histochemically. Upon harvesting, roots were washed, cleared in 10% potassium hydroxide at 4°C overnight. After clearing, root samples were washed three times with deionized water, acidified with 2% acetic acid for 5 min at room temperature and stained by immersion in a solution of 5% ink (Lamy, Germany) and 2% acetic acid overnight. The excess of ink solution was removed by rinsing the root samples three times with deionized water (García et al., 2020). Mycorrhizal colonization was evaluated by quantifying the percentage of root length colonized by the fungus according to the grid line intersection method (Giovannetti & Mosse, 1980) under stereo microscope Motic SMZ. The percentage of root length colonized by *R. irregularis* was 83% ± 4%.

### Herbivory bioassay

After 6 weeks of sowing, inoculated and Ni plants were challenged with *S. exigua*. Larvae were reared on artificial diet (Elvira et al., 2010) without antibiotics until they reached the L3 instar. Eight 6‐week‐old plants per treatment were used. The fourth true leaf of each plant was detached using a scalpel. To prevent leaf desiccation, leaves were placed in individual petri dishes containing a filter paper which was moistened with 3 mL of sterile water every 2 days during the bioassay. Each leaf was then infested with two third‐instar *S. exigua* larvae. Petri dishes with the infested leaves were maintained in laboratory for 10 days under the same conditions of temperature, photoperiod and humidity as described in section 2.2 (*plant material and growing conditions*), but without direct exposure to sunlight. The weight of the larvae was recorded every 2 days. At the end of the bioassay, surviving larvae were collected and preserved at −80°C until further processing.

### DNA extraction and sequencing

Collected *S. exigua* larvae, 44 individuals in total, were surface disinfected with 75% ethanol for 90 s, rinsed with sterile water and preserved at −80°C until dissection. The whole gut, including the gut content, was dissected, and the total DNA was extracted using the DNeasy Blood & Tissue Kit (Qiagen), following the manufacturer's instructions with few modifications. In brief, lysis reaction of the samples with Proteinase K was left overnight and final elution volume (buffer AE) was 100 μL. DNA extracts were stored at −20°C until further processing.

DNA amplification was conducted using the pair of primers S‐D‐Bact‐0341‐b‐S‐17 (5′‐CCTACGGGNGGCWGCAG‐3′) and S‐D‐Bact‐0785‐a‐A‐21 (5′‐GACTACHVGGGTATCTAATCC‐3′) (Herlemann et al., 2011). DNA libraries were generated using a limited cycle PCR: initial denaturation at 95°C for 5 min, followed by 25 cycles of annealing (95°C 40 s, 55°C 2 min, 72°C 1 min) and extension at 72°C for 7 min. After DNA amplification, Illumina sequencing adaptors (*Nextera XT* index kit v2, 600 cycles 300 × 300) were added to the amplicon. Libraries were then normalized, pooled and sequenced at the Sequencing Center of Ludwig‐Maximilian, University of Munich (Germany).

### Metabarcoding library filtering and taxonomic classification

Firstly, quality of the libraries was checked using *FastQC* (Andrews, 2010). Primers were removed, and forward and reverse reads were merged with minimum *Phred* score of 30 using *cutadapt* (Martin, 2011) and *PEAR* (Zhang et al., 2014) software programs, respectively. The software *vsearch v2.8.2* (Rognes et al., 2016) was used to conduct the subsequent filtering steps. Reads with more than one nucleotide mistake were discarded (*fastq_maxee* = 1) and only unique sequences were kept. This step was followed by the removal of potential insertions and deletions and chimeras (‘de novo’). At this point, we obtained the amplicon single variants (ASVs) that corresponded to our bacterial communities, which were stored in a *FASTA* file. Sequences were then classified against *Silva database v138.1* (Quast et al., 2012) using the *mothur* software (Schloss, 2020; Schloss et al., 2009).

The ASV table was filtered in ‘R’ software (R Core Team, 2023), using package *phyloseq* (McMurdie & Holmes, 2013) to remove contaminant sequences (unassigned, chloroplast and mitochondria). Six samples that had over three times lower or higher number of reads than the average of their treatment were also removed (Table S3).

### Data analysis

#### Biodiversity analyses on gut bacterial communities

To measure the completeness of our sampling, we evaluated the total richness of gut bacterial communities of *S. exigua* using a rarefaction curve and a bootstrap estimator with the function *specpool* in the *vegan* R package (Oksanen et al., 2013). For each treatment, coverage‐based rarefaction and extrapolation curves and sample size‐based for ASVs richness or exponential of Shannon index entropy, hereinafter *Shannon diversity*, were generated for each treatment using *iNEXT* R package (Hsieh et al., 2016). ASV richness and Shannon diversity were compared between treatments using ANOVA followed by Tukey's HSD test due to unbalanced larvae number across treatments after filtering steps. This analysis was performed using the *HSD*.*test* function of R package *agricolae* (De Mendiburu, 2023). The Shapiro–Wilk and Levene's tests were previously used to test the ANOVA assumptions. Functions selected are included in *car* and *stats* R packages.

Shared among treatments and treatment‐specific gut bacteria of *S. exigua* were addressed with the *microbiome* R package. As a rule, for one ASV to be present in a treatment, it must have an 85% prevalence and a minimum detection threshold of 0.1% of relative abundance. Relative read abundance of bacterial genera across treatments was computed using a customized script (*dplyr* R package) (Wickham et al., 2021). Separations among treatments were visualized using a principal coordinates analysis (PCoA) ordination plot based on Bray–Curtis dissimilarity matrix generated with *vegan* and *ggplot2* (Wickham, 2011). Permutational multivariate analysis of variance (PERMANOVA, 999 permutations) and analysis of multivariate dispersions were used to test the effect of root microbial inoculations on *S. exigua* gut bacterial community composition. Functions required to perform these analyses are implemented in the *vegan* R package. Then, differences between treatments were assessed using *pairwiseAdonis* R package (Arbizu, 2019).

Then, R package *microeco* (Liu et al., 2021) was used to identify the differential abundances of ASVs driving the significant dissimilarities between treatments (microbial inoculants). Linear discriminant analysis effect size (LEfSe) was applied, and ASVs displaying a LDA score ≥3.5 were selected for further analyses.

#### *Functional prediction of* S. exigua *gut bacteria*

The software ‘Phylogenetic investigation of communities by reconstruction of unobserved states’ (*PICRUSt2*) was used to predict functions from the 16S rRNA gene amplicons (Douglas et al., 2020). The analysis was performed on the ASVs of *S. exigua* gut bacterial core. The metabolic pathways to functions were predicted using the *MetaCyc* database (Caspi et al., 2020). Different classification levels (i.e., metabolic parental classes, superclasses and pathways) were considered when exploring the potential functions associated to *S. exigua* gut core bacteria.

#### Statistical analyses

All statistical analyses were conducted using the ‘R’ software. Graphs were generated using *ggplot2* and *ggpubr* (Kassambara, 2023) R packages. To analyse the impact of the treatments on *S. exigua* weight a linear mixed‐effect model (lmer function in the lmerTest package, Kuznetsova et al., 2017), the microbial treatments as fixed factor and the initial larval weight as random factor was used: lmer(FinalWeight ~ Treatment + (1|InitialWeight)). The different microbial treatments were compared to the control (Ni) treatment with multiple comparisons of means using the multcomp package (Hothorn et al., 2016). To investigate the connection between *S. exigua* bacterial community and insect biomass, Spearman's rank correlation and ENVfit analyses were performed. For that, we used the function *cor.test* of *stats* R package and the function *envfit* of *vegan* R package, respectively.

## RESULTS

### *Impact of plant beneficial microbes on* S. exigua *gut bacterial diversity*

Gut bacterial communities of *S. exigua* larvae were compared among treatments to investigate possible changes linked to different plant microbial inoculations—control (Ni), fungal (Ri or Th) or bacterial (Ba or Pa) inoculants. We obtained 2,126,658 raw reads from 16S rRNA gene sequencing of 44 *S. exigua* gut samples. After filtering six samples exhibiting three times less or more reads than their treatment average, 38 individuals were retained and used for further analysis (Table S1). No significant differences in the number of reads between treatments were found after excluding these six samples (*F* = 2.34, *p* = 0.08). Species richness estimation (based on a bootstrap analysis) suggested that our sampling captured 90.35% of the total diversity of the gut bacterial communities of *S. exigua* (Figure S1). Shannon diversity‐based (Figure S1) and coverage‐based curves for each treatment (coverage ≥95% for all treatments, Figure S1) did reach the plateau, assessing that the sequencing depth was sufficient to provide an adequate representation of *S. exigua* gut bacterial communities.

**FIGURE 1:**
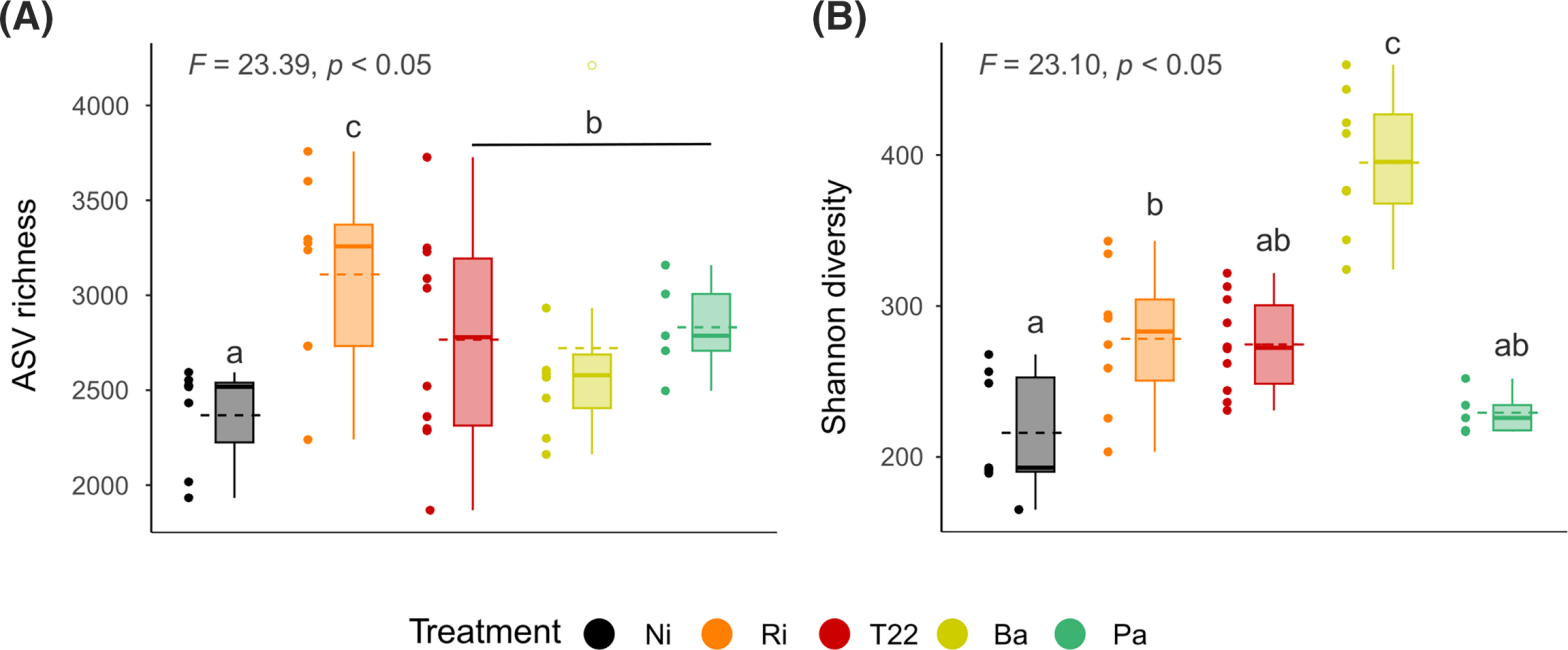
Alpha diversity of *Spodoptera exigua* across microbial treatments. (A) Amplicon single variant (ASV) richness and (B) Shannon diversity of caterpillars fed on non‐inoculated plants (Ni) or plants inoculated with *Rhizophagus irregularis* (Ri), *Trichoderma afroharzianum* T22 (T22), *Bacillus amyloliquefaciens* (Ba) or *Pseudomonas azotoformans* (Pa). In boxplot, boxes represent the interquartile range; solid bold lines represent the median; dashed lines crossing the boxplots represent the mean value for the treatment; whiskers represent maxima and minima within 1.5 times the interquartile range; and empty dots represent outliers. Values for microbial inoculation effect on alpha diversity measures according to ANOVA are presented at top left of each panel. Treatments not sharing a letter are statistically different based on Tukey's HSD test for unbalanced sample size.

Both ASV richness and Shannon diversity were affected by root microbial inoculations (*F* = 23.39, *p* <0.05 and *F* = 23.10, *p* <0.05, respectively; see Figure 1A,B).

Regarding the former metric, the number of reads of each sample was added as a cofactor in the model, as these parameters showed collinearity (Figure S2). This correlation was not found for Shannon diversity‐sample reads (*rho* = 0.07, *p* = 0.68). While all microbial treatments affected the alpha diversity of *S. exigua* gut bacteria by increasing the number of ASVs compared to control plants, Ri and Ba also increased the Shannon diversity (Figure 1A,B).

### *Impact of soil‐borne microbes on* S. exigua *gut bacterial composition*

**FIGURE 2:**
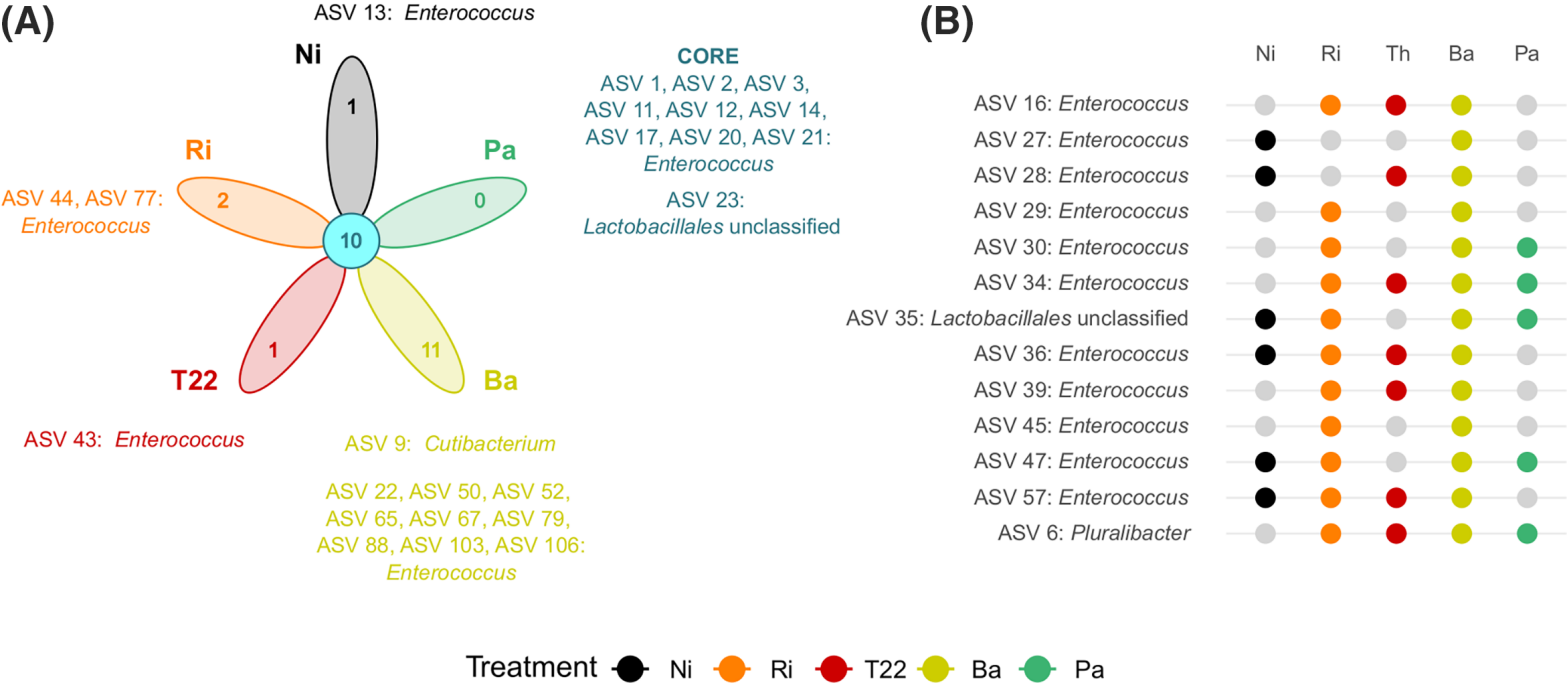
Common and treatment‐specific *Spodoptera exigua* gut bacteria. Amplicon single variants (ASVs) present in caterpillars fed on plants inoculated with different microbial treatments and their taxonomic assignment at the genus level. (A) ASVs common to all treatments (centre of the flower plot, in blue) and ASVs specific to caterpillars fed on a particular treatment (petals, colours correspond to microbial inoculations). (B) ASVs common to 2–4 microbial treatments. Caterpillars fed on non‐inoculated plants (Ni) or plants inoculated with *Rhizophagus irregularis* (Ri), *Trichoderma afroharzianum* T22 (T22), *Bacillus amyloliquefaciens* (Ba) or *Pseudomonas azotoformans* (Pa). In panel B, ASVs (rows) present in a treatment are shown as a coloured dot. Each colour represents one treatment (columns).

Among all analysed ASVs, 10 were shared by all *S. exigua* larvae regardless of root inoculations, while another set of ASVs were specifically associated to larvae fed on plants inoculated with certain beneficial microbes (Figure 2A,B).

Ba treatment presented the higher number of specific bacterial members, while Pa presented the lowest (Figure 2A). Most either shared or treatment‐specific ASVs belonged to the genus *Enterococcus* (Figure 2A,B), except for ASV 23, shared by all caterpillars and assigned to order *Lactobacillales* and ASV 9 (*Cutibacterium* spp.), specific for Ba‐fed larvae. ASV 6, assigned to genus *Pluralibacter*, was shared by larvae fed on inoculated plants, but not for those fed on control plants.

**FIGURE 3:**
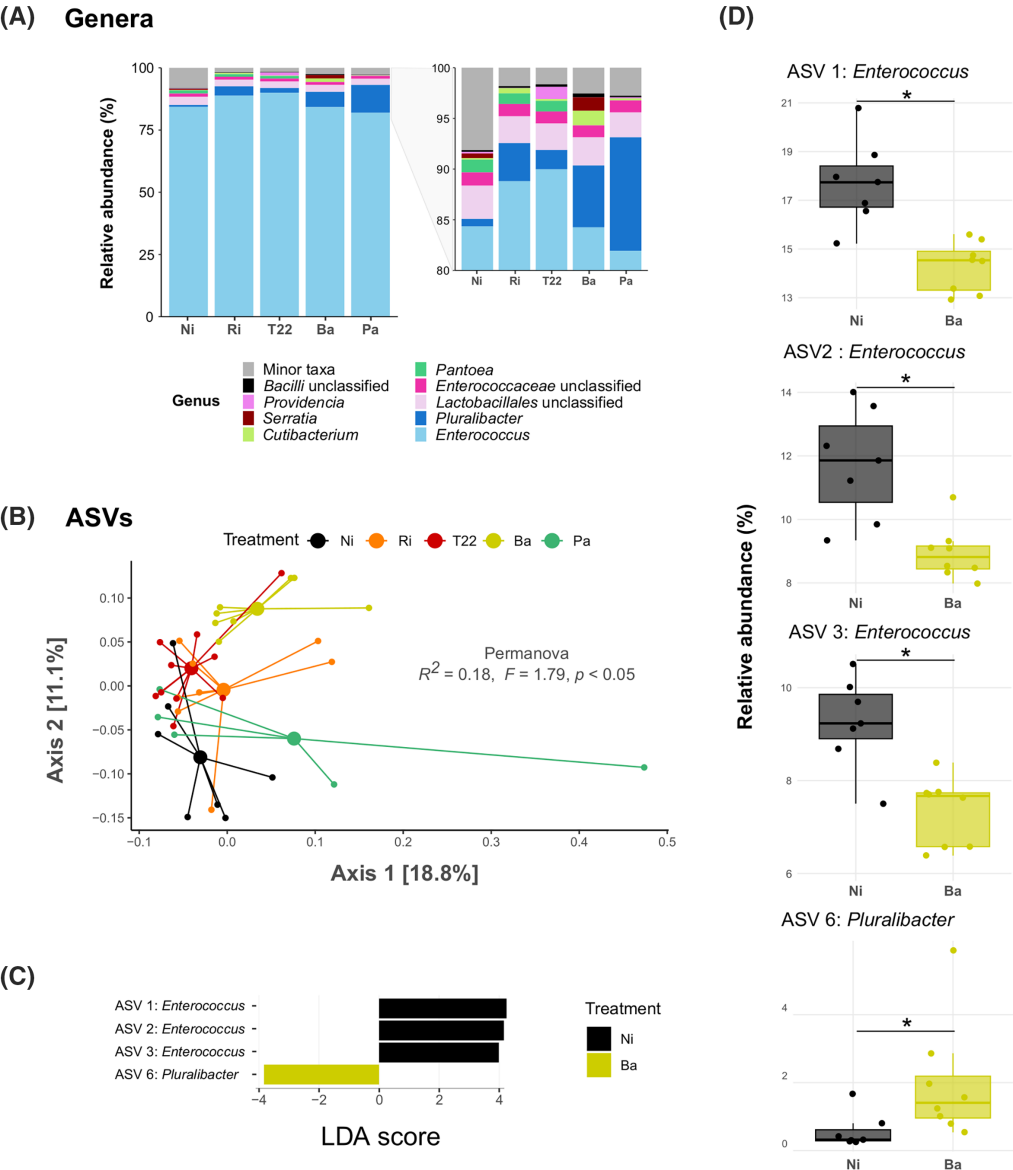
Gut bacterial community composition of *Spodoptera exigua* according to microbial treatments. (A) Relative abundance of top *S. exigua* gut bacteria and zoom‐up of less abundant taxa. (B) Principal Coordinates Analysis calculated with Bray–Curtis dissimilarity. (C) Differentially abundant amplicon single variants (ASVs) in caterpillars fed on non‐inoculated plants (Ni) or plants inoculated with *Bacillus amyloliquefaciens* (Ba) according to linear discriminant analysis effect size (LefSe). ASVs displaying a LDA score higher than 3.5 are shown. (D) Comparison of relative abundances of ASVs obtained in LefSe analysis between larvae fed on Ni plants and Ba plants. Plants inoculated with *Rhizophagus irregularis* (Ri), *Trichoderma afroharzianum* T22 (T22) or *Pseudomonas azotoformans* (Pa). In bar plots, genera accounting for lower relative abundance than the top 9 genera were merged into ‘Minor taxa’. In PCoA, small circles depict individual samples. Larger markers correspond to centroids. Percentage of variance explained by the axes is indicated between brackets. Significance of ‘microbial inoculation’ effect on *S. exigua* gut bacterial community based on a PERMANOVA is represented in the centre of the panel. In boxplots, boxes represent the interquartile range; solid bold lines represent the median for each treatment; whiskers represent maxima and minima within 1.5 times the interquartile range; and empty dots represent outliers. Asterisks represent statistical differences according to Student's *t*‐test.

*Firmicutes* was the most abundant phylum, accounting for 91.4% of the total analysed reads, followed by *Proteobacteria* (6.9%), *Actinobacteriota* (1.2%) and minor represented phyla (0.5%). Microbial treatments did not induce differences at this taxonomic level nor in terms of bacterial genera composition. Enterococci were the predominant bacteria in *S. exigua* guts, comprising 81%–89.9% of the relative abundance independently of microbial treatments, and the proportion of the rest of genera was variable (Figure 3A,B).

At ASV level, gut bacterial composition was significantly affected by plant‐associated microbes (*R* ^2^ = 0.15, *F* = 1.26, *p* <0.05; Figure 3C). The homogeneity of dispersions between treatments was confirmed (*F* = 0.61, *p* = 0.68), thus assessing that differences between the centroids distances were due to microbial treatment with no interference of the data dispersion in the results. Specifically, pairwise comparisons indicated that the community composition of caterpillars fed on Ba plants were different to those fed on Ni plants (Table S2).

Further analyses showed that three ASVs (ASV 1, ASV 2 and ASV 3), assigned to genus *Enterococcus*, and ASV 6, assigned to genus *Pluralibacter*, were differentially abundant when comparing caterpillars fed on control (Ni) plants and larvae fed on Ba plants (LDA score higher than 3.5; Figure 3C,D). The first three were ASVs common to all treatments, as previously shown in Figure 2A, while ASV 6 was specific for larvae fed on microbial‐inoculated plants but not for those fed on Ni (Figure 2B).

### *Impact soil microbes on* S. exigua *weight and potential connection between caterpillar performance and gut bacterial community alteration*

**FIGURE 4:**
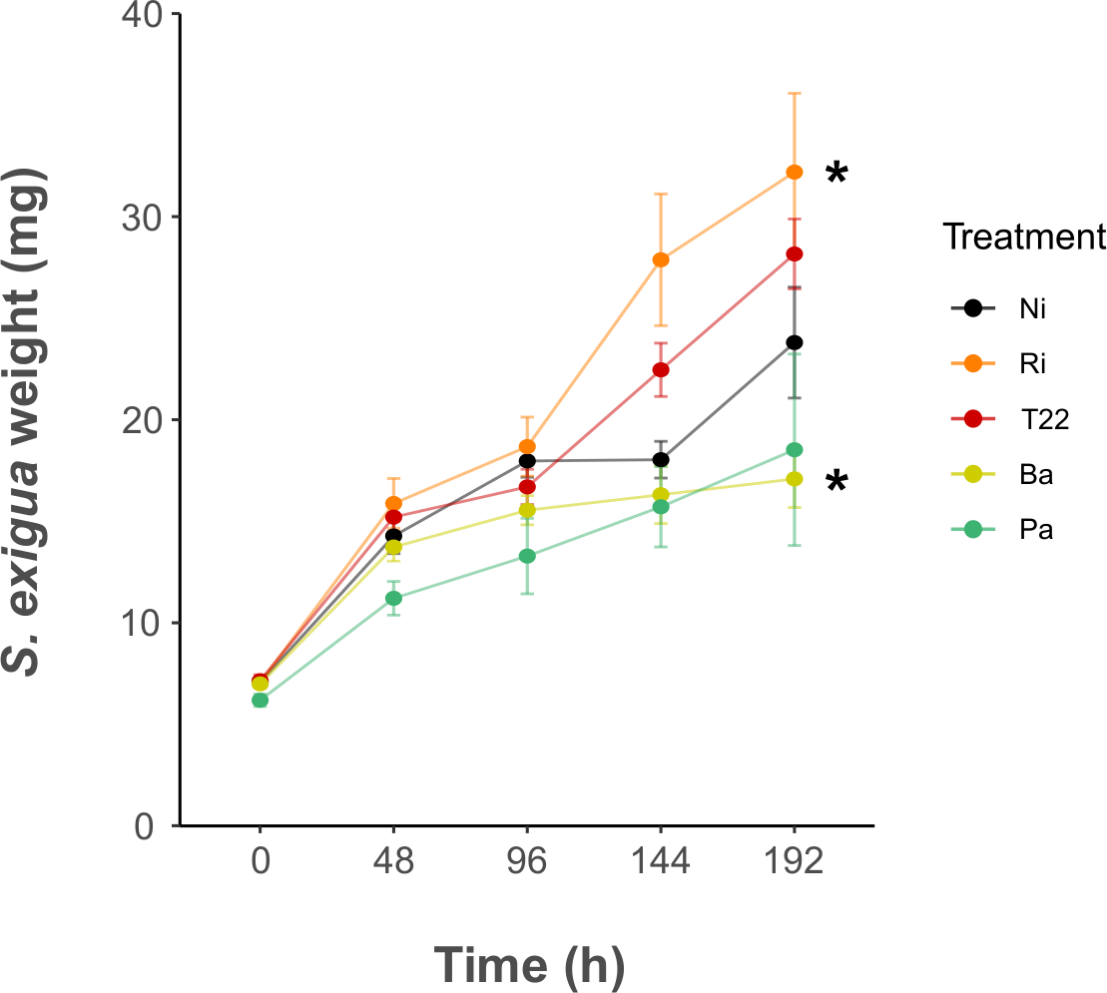
Impact of microbial treatments on *Spodoptera exigua* biomass. Non‐inoculated control plants (Ni), plants inoculated with: *Rhizophagus irregularis* (Ri), *Trichoderma afroharzianum* T22 (T22) *Bacillus amyloliquefaciens* (Ba) or *Pseudomonas azotoformans* (Pa). Asterisks represent statistical differences respect to control plants according to linear mixed‐effect model followed by Multiple Comparisons of Means (*p <0.05, *n* = 8).

To evaluate the effect of microbial root inoculation on caterpillar performance, *S. exigua* weight gain was determined in larvae fed on detached leaves of tomato plants from the different treatments. Microbial inoculations were shown to influence this parameter (*F* = 5.73, *p* <0.05) differentially. Larval weight was significantly lower when feeding on leaves of plants inoculated with Ba as compared to Ni plants. On the contrary, inoculation with Ri increased caterpillar weight (Figure 4). Significant effects were not observed for larvae fed on T22 or Pa treatments (Figure 4).

**FIGURE 5:**
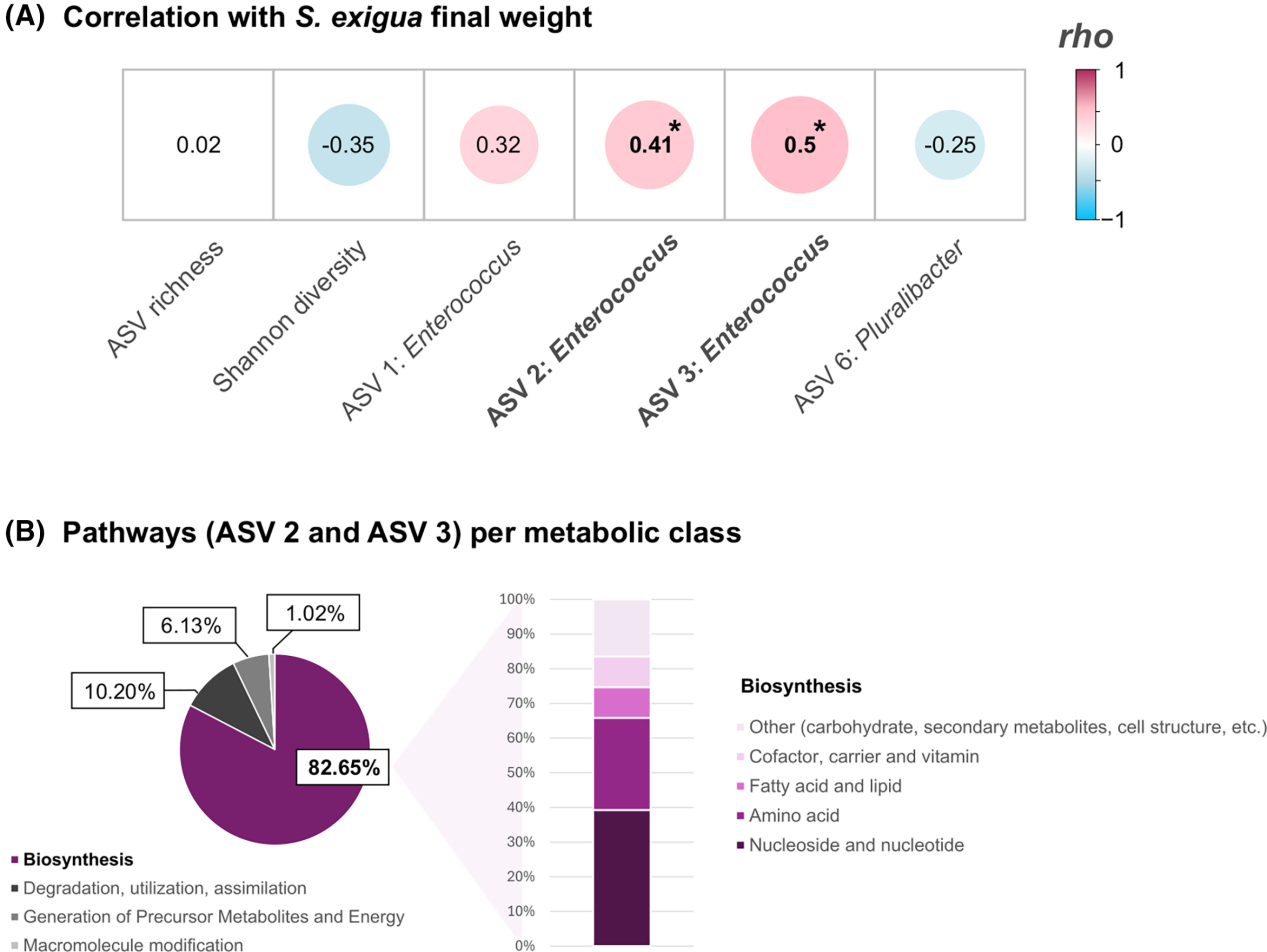
Correlation between *Spodoptera exigua* final weight and gut bacterial community, and functional prediction of amplicon single variants (ASVs) correlated with larval performance. (A) Correlation between *S. exigua* final weight and insect bacterial community. ASV richness and Shannon diversity were chosen as measures of alpha diversity. Four ASVs responsible for differences in *S. exigua* gut beta diversity were used for correlation analyses between insect performance and gut bacterial composition. Negative numbers (blue) represent negative correlations. Positive number (pink) represent positive correlations. The diameter of the circles represents the magnitude of the correlations. Asterisks (bold) indicate statistical significance according to Spearman's rank correlation analyses. (B) Metabolic pathways of ASV 2 and ASV 3 (*Enterococcus* sp.) predicted by *PICRUSt2* analysis. Pie chart represents the percentage of pathways associated to each different metabolic parent class according to *MetaCyc* database. Bar graph represents the percentage of pathways associated to each ‘Biosynthesis’ (the most represented parent class) superclass according to *MetaCyc* database.

To assess the potential connection between changes induced by root inoculations in the gut bacteria community of the larvae and the distinct performances observed, the parameters affected were subjected to correlation analysis. Two of these parameters, related to *S. exigua* microbiota composition significantly correlated with larval performance. Specifically, the relative abundance of ASV 2 and ASV 3, assigned to *Enterococcus* spp., exhibited positive correlations with the biomass of the insects (Figure 5A). Furthermore, the envfit outcome supported these results, pointing to a significant effect of microbial treatments (*R* ^2^ = 0.29, *p* <0.05) on both, the bacterial community of caterpillars as well as on their final weight (*R* ^2^ = 0.17, *p* <0.05).

Then, metabolic functions for ASV 2 and ASV 3, displaying higher relative abundance in Ni‐fed larvae than in Ba‐fed larvae and positively correlated with larval weight, were predicted using PICRUSt2. We found that most predicted pathways—the 82.65% (Figure 5B, pie chart)—corresponded to the ‘Biosynthesis’ parent class according to MetaCyc database, followed by pathways associated to metabolic degradation/ utilization/ assimilation (10.20% of the total; Figure 5B, pie chart). The 6.13% of pathways were related to precursor metabolites and energy generation, and the rest corresponded to macromolecule modification (Figure 5B, pie chart). We focused on the parent class biosynthesis for further analyses, as it contained the vast majority of metabolic pathways predicted.

More than the 65% of biosynthesis pathways corresponded to two main functional subclasses, ‘Nucleotide/nucleoside biosynthesis’ and ‘Amino acid biosynthesis’ (Figure 5B, bar plot zooming‐up the pie chart area corresponding to parental metabolic class ‘Biosynthesis’). These categories were followed by ‘Cofactor, carrier and vitamin biosynthesis and fatty acid and lipid biosynthesis’ (8.86% of total predicted paths each one; see Figure 5B bar plot). The remaining subclasses (i.e., carbohydrate or secondary metabolite biosynthesis) accounted for approximately the 16.5% of total predicted pathways (Figure 5B, bar plot). Thus, functional prediction of bacterial ASVs correlated with *S. exigua* performance was characterized by a high prevalence of pathways associated to ‘Nucleotide/nucleoside biosynthesis’ and ‘Amino acid biosynthesis’. Among the top 15 most abundant biosynthesis paths, we found some of them associated to biosynthesis of amino acids, nucleosides/nucleotides, fatty acids and lipids or cofactors/carriers/vitamins (Figure S3). Major number of top pathways (almost 50% of the total) corresponded to ‘Amino acid biosynthesis’. Remarkably, the number of paths related to biosynthesis of the essential amino acids (i.e., Val, Met and specifically Ile) was particularly high. All predicted functions were more abundant in guts of *S. exigua* larvae fed on Ni plants compared to larvae fed on Ba plants, coinciding with the better performance of the first group of caterpillars (Figure 5C).

## DISCUSSION

Our study reveals that single species of root‐associated microorganisms can trigger plant‐mediated modifications of the gut bacterial community of the generalist leaf‐chewer *S. exigua*. The impact on diverse microbiota‐related parameters were inoculant‐specific, and we aimed to address whether those alterations were reflected in larval performance.

Several studies have reported that the microbiota of lepidopteran insects depends on their diet (Martínez‐Solís et al., 2020; Phalnikar et al., 2018; Tang et al., 2012), while others have shown little or no association (Chaturvedi et al., 2017; Whitaker et al., 2016). A recent study demonstrated that foliar‐feeding caterpillars acquire their gut microbiota directly from the soil rather than from the host plant (Hannula et al., 2019). In this study, we aimed to test if soil‐borne beneficial microorganisms can alter the gut microbiota of aboveground caterpillars via bottom‐up plant‐mediation. We, therefore, used detached leaves to avoid any contact with the soil. We characterized the gut bacterial communities of *S. exigua* larvae fed on detached leaves of plants that were inoculated or not in their roots with single species (monoassociations) of bacteria or fungi, with reported ability to stimulate plant defences (Minchev et al., 2021).

In agreement with previous studies on lepidopterans, *S. exigua* gut microbiota showed low diversity at all taxonomic levels and major contribution to relative read abundance was due to few bacterial genera (Chen et al., 2016; Ramírez‐Serrano et al., 2022; Xiang et al., 2006). At the ASV level, gut bacteria diversity was affected by microbial inoculations. All tested inoculants contributed to richness augmentation, but Shannon diversity only increased in larvae fed on *R*. *irregularis* (Ri)‐ and *B*. *amyloliquefaciens* (Ba)‐treated plants. The second was the only inoculant that modified *S. exigua* bacterial community composition. We used two beneficial fungi, *T. afroharzianum* T22 (T22), besides the AMF, and two PGPR, *P. azotoformans* (Pa), besides Ba. Therefore, our results indicate that gut bacterial communities reshape depends on the microbial species inoculated rather than on higher taxonomic ranks (i.e., Eukaryotes vs. Prokaryotes) or ‘artificial’ classifications based on benefits and other mutualism‐related characteristics (i.e., PGPR), as we find both inter‐ and intra‐group variation.

Root colonization by rhizospheric microorganisms may have important effects on the microbial community composition of the phyllosphere (Debray et al., 2022; Gong & Xin, 2021; Poosakkannu et al., 2017). Since leaf‐associated bacteria are commonly found in insect gut microbiomes (Hammer et al., 2017; Hansen & Enders, 2022; Mogouong et al., 2021), changes induced by soil beneficial microbes in microbial populations of the phyllosphere might contribute to reshaping the gut microbiota of foliar‐feeding insects. Additionally, defensive and nutritional plant traits can affect microbiota already established in the insect (Hu et al., 2022; Mason et al., 2019). For instance, some plant defensive compounds have a dual antimicrobial and insecticidal activity (Al‐Khayri et al., 2023), and it is plausible that the latter could stem from the former. In essence, the insecticidal effect may be the ultimate consequence of the antimicrobial impact exerted by these compounds on the microbiome of the consuming herbivore (Hammer & Bowers, 2015). Therefore, the plant‐mediated variations in *S. exigua* microbiota observed in this study might reflect gut bacteria adaptations to different phyllospheric microbial inputs, or their responses to physiological metabolic plant changes induced by rhizospheric microbes. It remains to be investigated how soil microbes changed plant nutrition, defence and phyllospheric bacterial communities to address why some of them altered *S. exigua* gut bacterial composition while others did not.

Insect microbiota has been proposed to contribute to insect performance in different systems, but their role in lepidopterans is unclear (Frago et al., 2020; Kaiser et al., 2010; Sugio et al., 2015; Voirol et al., 2018). Whether their microbiomes contribute to the observed performances remained to be determined, and stablishing a potential correlation between both parameters was one of our objectives.

We show that the tested rhizospheric microorganisms have a different impact on *S. exigua* performance. Ba reduced larval biomass, while Ri increased it and neither T22 nor Pa affected it. Impaired larval performance associated with microbiota dysbiosis has led to suggest a potential metabolic exchange between caterpillars and their associated microorganisms (Broderick et al., 2006; Chen et al., 2020, 2022; Wang et al., 2020). In this study, root inoculation was found to cause different effects on both *S. exigua* gut microbial communities and performance depending on the rhizospheric microbial identities. Microbes with a greater impact on caterpillar bacterial community also changed insect performance. However, we observed correlations only between the specific changes that Ba caused on *S. exigua* bacterial composition and the performance of the insect. Benefits conferred to plants by microbes depends on the identities of both partners (Van Loon, 2007; Van Oosten et al., 2008) and are subjected to the environmental conditions (Bennett & Groten, 2022; Lee Díaz et al., 2021; Pineda et al., 2013). In the case of mycorrhiza, fine‐tuned regulation of the plant growth‐defence equilibrium has been proposed to determine plant prioritization for enhanced growth or resistance according to nutrient availability (Bennett & Groten, 2022; Dejana et al., 2022). It is therefore possible that under our experimental conditions Ri plants offered a better diet quality for *S. exigua*, considering that usually AMF confer nutritional benefits to the host plant. Ri‐fed caterpillars might have acquired more nutrients directly from their diet, leading to increased larval biomass despite not displaying significant changes in their bacterial composition. Previous research has documented varying weight gain of *S. exigua* on mycorrhizal plants regardless of caterpillar bacterial community, associated with differences in the plant nutritional status (Ramírez‐Serrano et al., 2022). Additional experiments are required to prove this hypothesis.

*Bacillus amyloliquefaciens* changed the relative abundance of several gut bacteria previously reported in insects fed on plant diets (Enya et al., 2007; Micallef et al., 2013; Patel et al., 2017; Xu & Kim, 2014). Specifically, two enterococcal ASVs positively correlated with *S. exigua* biomass were less abundant in caterpillar fed with Ba‐inoculated plants. It has already been demonstrated that a single soil‐borne microbial species can affect the enterococci populations of larvae of the genus *Spodoptera* (Di Lelio et al., 2023). It has also been observed that specific enterococci can drastically affect the growth of different species within this genus of insects (Di Lelio et al., 2023; Mason et al., 2022). Moreover, enterococci‐mediated improvement of caterpillar performance has been reported as dependent on the diet quality (Chen et al., 2022). Specifically, these bacteria seem to be of particular importance when the diet is suboptimal (Chen et al., 2022).

In the case of the mutualism between *Trichoderma afroharzianum*‐tomato, the diet‐dependent relationship between *Spodoptera littoralis* enterococcal symbionts and insect performance has been associated with a reduced nutritional support to the insect host (Di Lelio et al., 2023). The authors of this study showed that the transcription of metabolic pathways associated with biosynthesis of essential amino acids and sugars was reduced in larvae fed on plants inoculated with a soil beneficial fungus. Consequently, these caterpillars displayed an impaired growth and development compared to larvae fed on non‐inoculated plants (Di Lelio et al., 2023). Our findings are in agreement with these observations. We focused on the two enterococcal ASVs showing a lower prevalence in larvae fed on Ba‐inoculated plants compared to Ni‐fed caterpillars. We observed a positive correlation between the abundance of these ASVs and *S. exigua* performance, and we made a functional prediction for these bacterial taxa. Then, we found that the Ba‐mediated reduced abundance of specific enterococci, potentially associated with a lower biosynthesis of important metabolites such as essential amino acids, might be responsible of *S. exigua* growth impairment in Ba plants. Furthermore, the importance of amino acids for caterpillars' growth was also observed by Šigutová et al. (2024) in a different model system. The authors found that a decreased performance of caterpillars fed on an artificial diet supplied with plant defence metabolites was also related to lower amino acid metabolism (Šigutová et al., 2024).

Overall, our results show that plant symbionts, indirectly through the plant, can differentially influence the gut bacterial community of *S. exigua*, as well as the performance of the insect. Our findings suggest that the association between the altered larval microbiota and performance may not result solely from the inoculated microorganism's ability (mediated by the plant) to modify the insect bacterial community. Instead, specific alterations in the caterpillar microbiome and plant defensive and nutritional traits induced by soil‐borne microbes might have occurred. In summary, this study illustrates the complexity and specificity of multiway interkingdom interactions, shedding light on additional mechanisms by which soil beneficial microorganisms can aid plants in protecting against insect pests.

## AUTHOR CONTRIBUTIONS

**Beatriz Ramírez‐Serrano:** Writing – original draft; writing – review and editing; formal analysis; methodology; data curation; conceptualization. **Marina Querejeta:** Data curation; writing – review and editing; formal analysis; methodology. **Zhivko Minchev:** Methodology; writing – review and editing. **María J. Pozo:** Resources; supervision; writing – review and editing. **Géraldine Dubreuil:** Conceptualization; writing – review and editing; supervision. **David Giron:** Funding acquisition; conceptualization; writing – review and editing; project administration; supervision; resources; writing – original draft.

## CONFLICT OF INTEREST STATEMENT

The authors declare no conflicts of interest.

## Supporting information


**Data S1.** Supporting information.

## Data Availability

The raw dataset generated and analyzed during the current study is openly accessible in the NCBI Sequence Read Archive (SRA) under the BioProject PRJNA1089387. The original contributions presented in the study are included in the article/Supplementary Material.
