# Supplementary material for: Root inoculation with soil‐borne microorganisms alters gut bacterial communities and performance of the leaf‐chewer Spodoptera exigua
Source: Environ Microbiol Rep. 2024 Nov 26;16(6):e70049. doi: 10.1111/1758-2229.70049 (PMC11598745; doi:10.1111/1758-2229.70049)
Supplement: Supplementary file 1 — Data S1. Supporting information. [file EMI4-16-e70049-s001.docx]

**Table S1. Primers for RT-qPCR for microbial DNA identification in rhizosphere samples.**

| Microorganism | Gene | Primers (5'- 3') | Reference |
| --- | --- | --- | --- |
| *Bacillus amyloliquefaciens* CECT 8238 | *Bamy6614_00315* | ACAAGGGTGGTTTATGGGCT  GCTCTCGGCCTGCAGATTAT | This study |
| *Pseudomonas azotoformans* | *RpoD* | AAGGACATCAACCGTCGCAT  CCGATGTTGCCTTCCTGGAT | This study |
| *Trichoderma harzianum* | *Tef-1𝛼* | GGTACTGGTGAGTTCGAGGCTG  GGGCTCGATGGAGTCGATAG | (Martínez-Medina et al., 2017) |
| *Rhizophagus irregularis* | *nLRS 28S* | TTCGGGTAATCAGCCTTTCG  TCAGAGATCAGACAGGTAGCC | (Thonar et al., 2012) |
| *Solanum lycopersicum* | *SlEF-1𝛼* | GATTGGTGGTATTGGAACTGTC  AGCTTCGTGGTGCATCTC | (Rotenberg et al., 2006) |

**REFERENCES**

**Martínez-Medina A, Appels FVW, van Wees SCM. 2017.** Impact of salicylic acid- and jasmonic acid-regulated defences on root colonization *by Trichoderma harzianum* T-78. *Plant Signaling and Behavior* **12**, 1-4. https://doi.org/10.1080/15592324.2017.1345404

**Rotenberg D, Thompson TS, German TL, Willis DK. 2006.** Methods for effective real-time RT-PCR analysis of virus-induced gene silencing. *Journal of Virological Methods* **138**, 49–59. https://doi.org/10.1016/j.jviromet.2006.07.017

**Thonar C, Erb A, Jansa J. 2012.** Real-time PCR to quantify composition of arbuscular mycorrhizal fungal communities-marker design, verification, calibration and field validation*. Molecular Ecology Resources* **12**: 219–232. <https://doi.org/10.1111/j.1755-0998.2011.03086.x>

**Table S2. Microbial relative DNA amount in tomato roots referred to plant DNA**

| Microbe | Microbial DNA relative to plant DNA | Standard error |
| --- | --- | --- |
| Ri | 1.72E+01 | 4.38E+00 |
| T22 | 9.75E-03 | 7.84E-03 |
| Ba | 8.48E-04 | 2.71E-04 |
| Pa | 5.18E-04 | 2.18E-04 |

Table S3. Sequencing information of samples across treatments

Grey italic letters indicate the caterpillars that were removed from the analyses according to filtering criteria (three times higher or lower number of reads than treatment average).

Table S4. Pairwise comparisons of bacterial composition within microbial treatments.

Pairwise PERMANOVA with Bray-Curtis dissimilarities (999 permutations) were used. Bonferroni method was selected for *p* value adjustment.


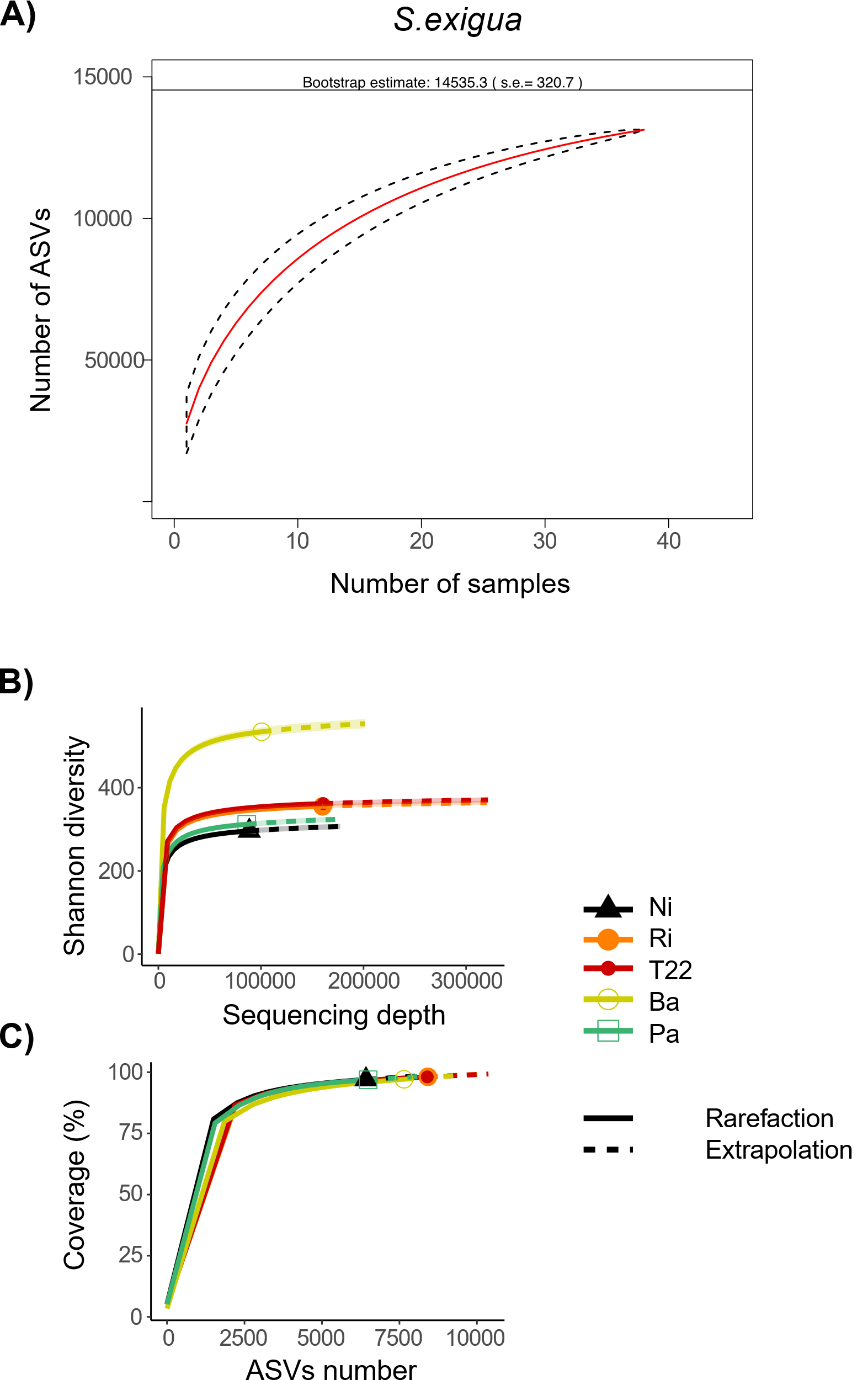


Figure S1. Rarefaction curves. (A) Rarefaction curve for ASV richness based on bootstrap estimate for *Spodoptera exigua* gut bacteria. (B) Sample size-based rarefaction curve of Shannon diversity for each treatment. (C) Coverage-based rarefaction curve of ASV richness for each treatment. Non-inoculated plants (Ni) or plants inoculated with *Rhizophagus irregularis* (Ri), *Trichoderma afroharzianum* T22 (T22), *Bacillus amyloliquefaciens* (Ba) or *Pseudomonas azotoformans* (Pa).


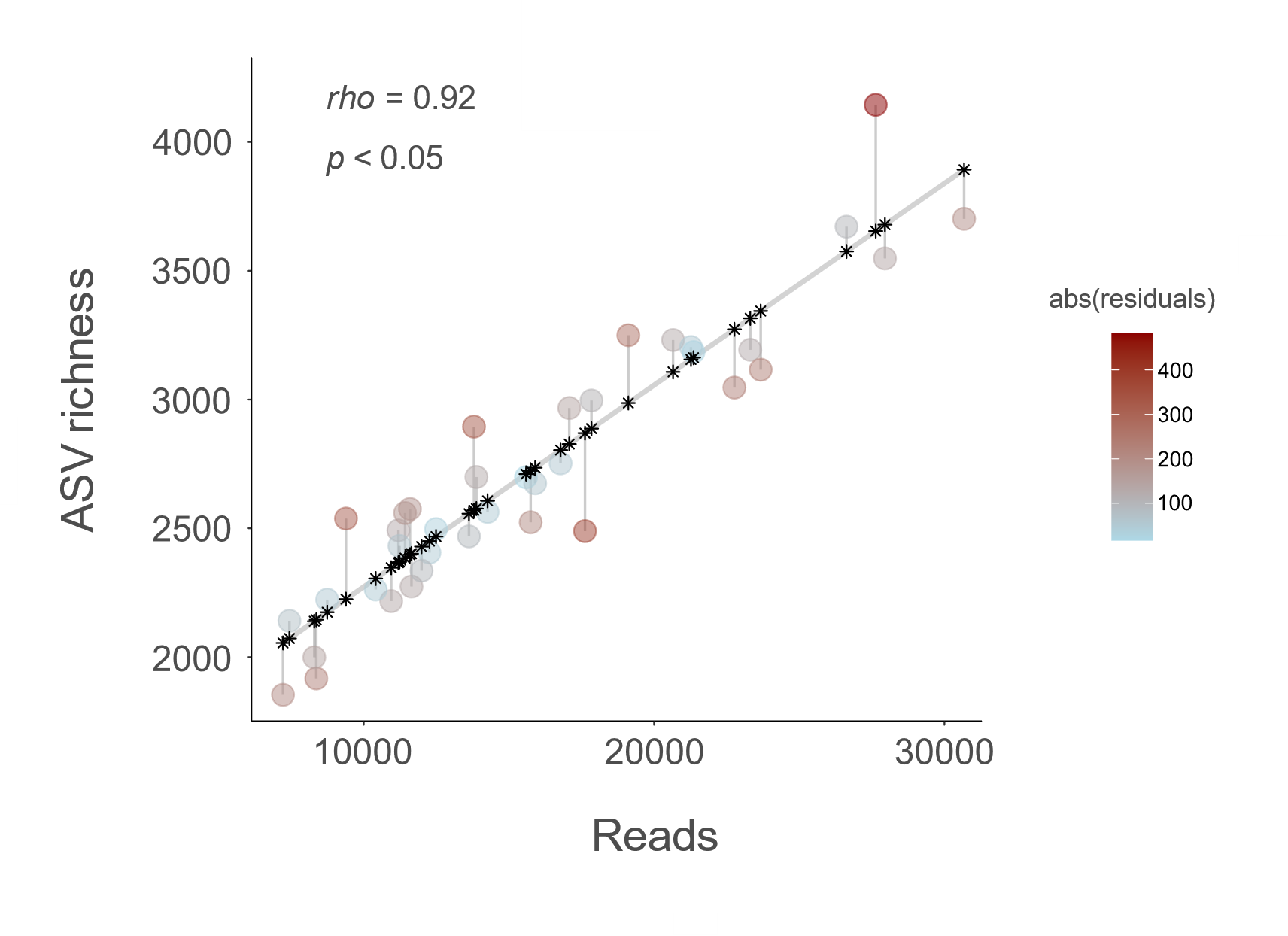


Figure S2. Correlation between ASV richness and sample reads. Predicted values of the linear model are represented as asterisks and residuals as points. Color legend represents the absolute value of the residuals.


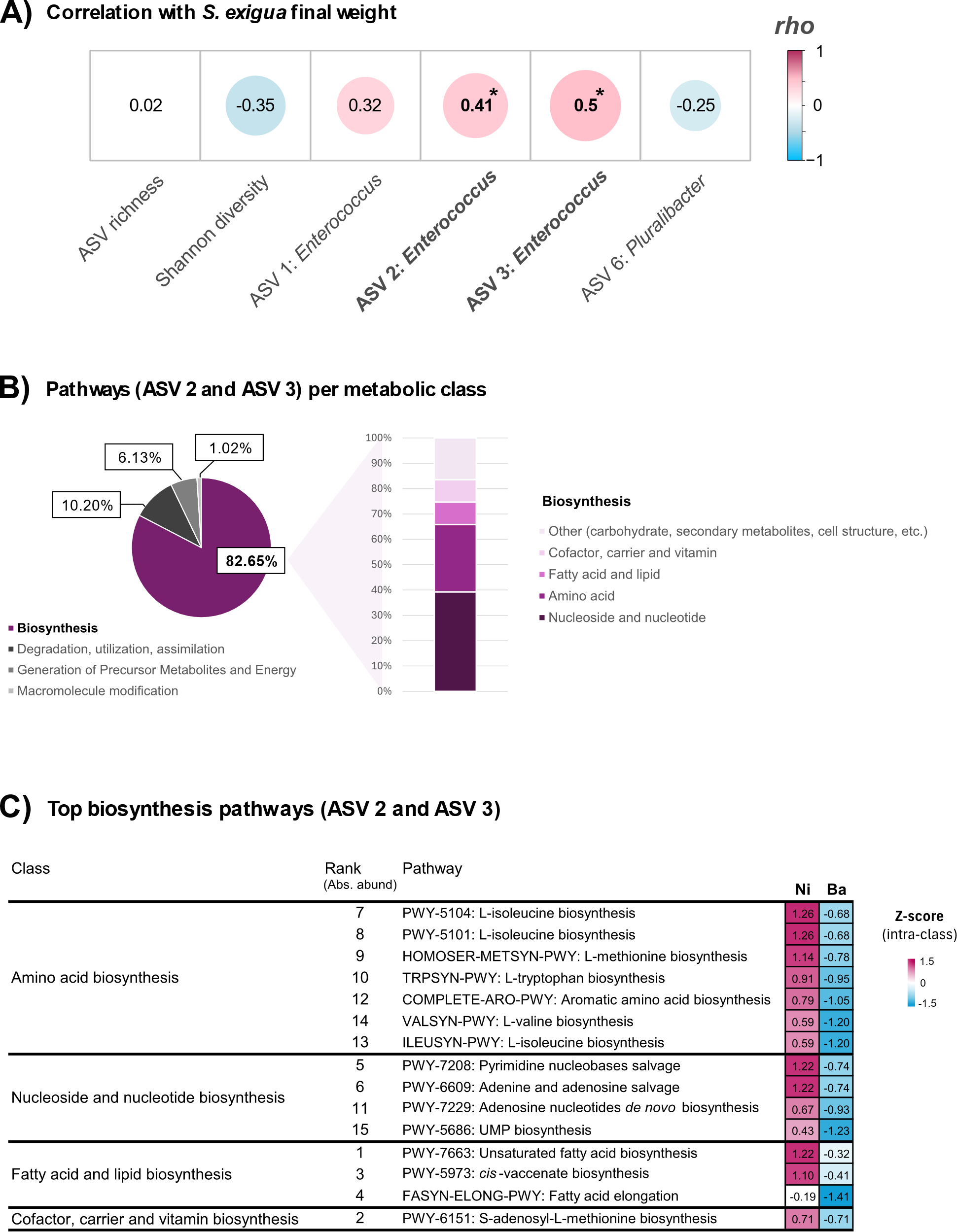


Figure S3. Top abundant biosynthesis pathways predicted by *PICRUSt2* for ASV2 and ASV 3. Rank values represent the rank of each pathway according to their absolute abundance (Abs. abund). Abundances of each top pathway in guts of *S. exigua* larvae fed on non-inoculated control plants (Ni) or plants inoculated with *Bacillus amyloliquefaciens* (Ba) are represented as Z-scores calculated for each metabolic class.
